# Acute Fulminant Viral Cerebellitis With Hypoxic-Ischemic Encephalopathy in a Male Child

**DOI:** 10.7759/cureus.69408

**Published:** 2024-09-14

**Authors:** Sheetal S Shelar, Shivali V Kashikar, Pratap Parihar, Mithun P Bhoyar, Rajasbala Dhande, Nirja Thaker

**Affiliations:** 1 Department of Radiodiagnosis, Datta Meghe Institute of Higher Education and Research, Wardha, IND

**Keywords:** acute cerebellitis, cerebellar herniation, fulminant, hypoxic-ischemic encephalopathy, viral

## Abstract

Acute cerebellitis is a rare inflammatory process affecting the cerebellum, commonly seen in the pediatric population following primary or secondary infection. Since the condition presents with a broad spectrum of clinical symptoms, radiological investigations, preferably magnetic resonance imaging, become essential in diagnosing it and planning further management. In this article, we discuss a case of a child presenting with a severe form of acute cerebellitis and hypoxic-ischemic encephalopathy secondary to brainstem compression.

## Introduction

Clinically, acute cerebellitis can manifest as a benign, self-limiting illness or as a potentially fatal, fulminant course [1,2]. The cause of acute cerebellitis remains unknown in about two-thirds of the cases. Meanwhile, the other third may be caused by bacteria or viruses, with viruses sometimes causing more severe symptoms and having a more severe course [3]. Common etiology includes varicella-zoster virus, Epstein-Barr virus, rubella, measles, herpes simplex virus, rotavirus, and echovirus [4]. A few cases have also been reported to be associated with the influenza virus. The relatively less severe form of acute cerebellitis is acute cerebellar ataxia, involving classical cerebellar findings like truncal ataxia, nystagmus, and tremors [5]. Sometimes, these signs may be absent with the presence of nonspecific symptoms like headache, fever, nausea, vomiting, altered mental status, and even coma [4,5]. The fulminant type of acute cerebellitis usually involves diffuse cerebellar swelling with increased pressure in the infratentorial compartment, causing compression of the brainstem, hydrocephalus, and downward or upward herniation of the posterior fossa structures [6-8].

Hypoxic-ischemic encephalopathy is usually encountered in neonates due to antenatal/perinatal insult. However, in older children or adults, it occurs following catastrophic events like drowning and asphyxia [9]. Compression of the brainstem containing the respiratory/cardiac centers can also result in hypoxia. These patients generally present to the hospital with a history of intubation and prolonged resuscitation.

## Case presentation

A five-year-old male was brought to the casualty by his parents, who complained of multiple episodes of convulsions (generalized tonic-clonic seizures) and vomiting, fever, slurred speech, inability to walk, and difficulty breathing for a week. There was no history of ingestion or inhalation of any toxins. He had completed a three-day course of antibiotics from a primary healthcare center. The child was in a state of altered consciousness and was assessed in the emergency department with a Glasgow Coma Score of 3 (eye-opening response: 1, verbal response: 1, motor response: 1). The patient had low oxygen saturation (SpO_2_ below 90%) and fluctuating blood pressure. Bilateral pupils were reactive, and there was no evidence of nystagmus. The child was administered antiepileptic, with lorazepam being the first drug given (dose of 0.1 mg/kg) and was repeated. However, the seizures were not controlled, and phenytoin was administered (dose of 15 mg/kg). The dosage of phenytoin was further to 20 mg/kg, after which the child stopped convulsing. He was then subjected to blood investigations and radiological imaging.

**Figure 1:**
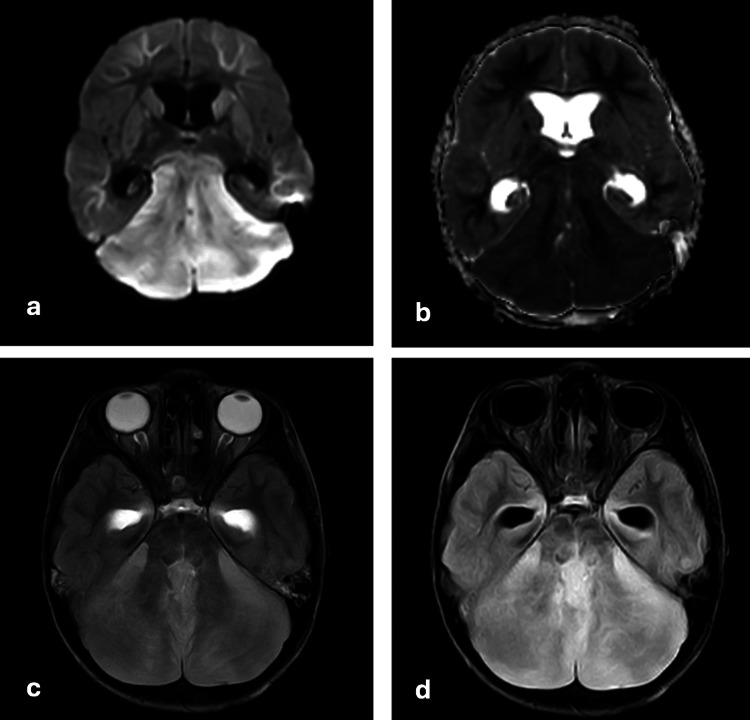
MRI brain axial sections depicting bilateral cerebellar hemispheres showing restricted diffusion on (a) DWI sequence with corresponding low signal intensity, (b) ADC sequence appearing homogenously hyperintense, (c) T2 showing evidence of increased T2 signal intensity around the bilateral optic nerves, and (d) FLAIR sequences MRI: magnetic resonance imaging; DWI: diffusion-weighted imaging; ADC: apparent diffusion coefficient; FLAIR: fluid-attenuated inversion recovery

**Figure 2:**
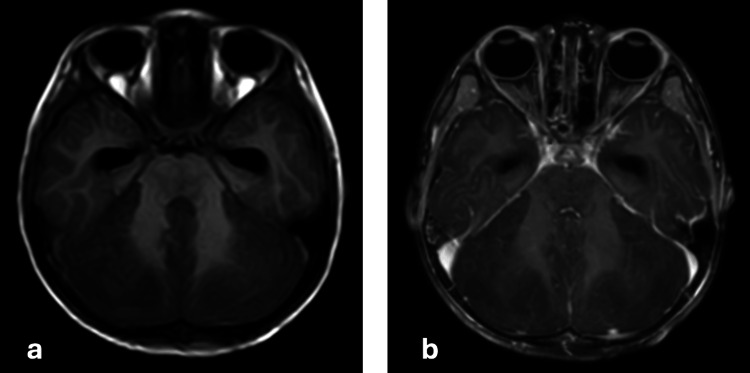
MRI axial T1-weighted (a) plain and (b) contrast images, showing no significant leptomeningeal or parenchymal enhancement MRI: magnetic resonance imaging

**Figure 3:**
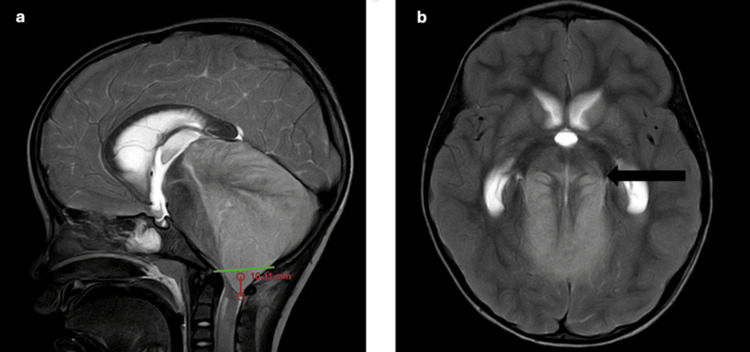
MRI brain T2-weighted (a) sagittal image showing the descent of the cerebellar tonsil by 10 mm from the McRae line and (b) axial image showing upward transtentorial herniation with obliteration of quadrigeminal cisterns and compression of the posterior aspect of midbrain giving “spinning top” appearance MRI: magnetic resonance imaging

**Figure 4:**
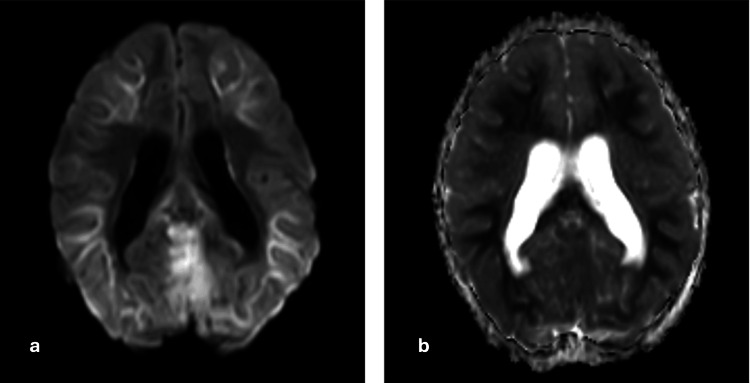
MRI brain axial sections (a) DWI and (b) ADC sequences showing diffusion restriction in the subcortical white matter of bilateral cerebral hemispheres. MRI: magnetic resonance imaging; DWI: diffusion-weighted imaging; ADC: apparent diffusion coefficient

On magnetic resonance imaging (MRI) of the brain with contrast, it was noted that bilateral cerebellar hemispheres showed restriction on diffusion-weighted imaging, with corresponding low signal intensity on apparent diffusion coefficient and hyperintensity on T2/fluid-attenuated inversion recovery sequences (Figure 1). There was also diffuse enlargement of the cerebellum, indicating cerebellar edema. Bilateral optic nerves appear edematous near the optic disc with high T2 signal intensity around it, suggesting bilateral papilledema. However, there was no evidence of significant leptomeningeal or parenchymal enhancement on contrast administration (Figure 2). There was downward cerebellar tonsillar herniation by 10 mm (measured from the McRae line connecting anterior and posterior margins of the foramen magnum) [10,11] and upward transtentorial herniation causing complete effacement of bilateral perimesencephalic cisterns and compression of midbrain anteriorly (Figure 3). There is complete effacement of the perimesencephalic cisterns and partial effacement of the fourth ventricle, causing mild hydrocephalus. Diffusion restriction was noted in the subcortical white matter of bilateral cerebral hemispheres, indicating changes in global hypoxic-ischemic encephalopathy (Figure 4).

The radiological features pointed toward a possibility of acute cerebellitis with changes in global hypoxic-ischemic encephalopathy. The hypoxic brain injury was thought to be a result of respiratory failure due to significant compression of the midbrain, pons, and medulla. Lumbar puncture was also performed for CSF analysis. On microscopy, CSF showed no RBC, pus cell, or organism (even on culture). Hence, the results pointed toward viral etiology. Since antibiotics serve no significant purpose in a viral infection, the child was managed by administering intravenous corticosteroids (methylprednisolone 30 mg/kg with a maximum dose of 1 mg/day) and supportive care. However, the condition of the patient did not improve in the following days, ultimately resulting in death.

## Discussion

Acute cerebellitis is one of the significant causes of acute cerebellar dysfunction in childhood, which could be either infectious (mostly viruses, as in our case) or post-vaccination [12-14]. It is a rare neurologic entity with variable clinical and imaging features [15]. Classical cerebellitis presents with cerebellar signs like ataxia, tremors, and nystagmus, whereas fulminant cerebellitis may not present these signs due to an altered level of consciousness in these patients [3]. Other differential diagnoses like cerebellar infarction and cerebellar tumors should be considered if only cerebellar signs are present. If the cerebellar signs are masked and not identifiable in sepsis, the possibility of meningoencephalitis must also be ruled out. Therefore, a combination of clinical and radiological information is always necessary to reach the final accurate diagnosis [16].

Computed tomography is useful only for detecting cerebellar edema, brainstem compression, or acute hydrocephalus. Even then, MRI is more sensitive than CT.

Initial symptoms in such patients could be acute onset fever with severe headache attributed to cerebellar swelling, causing an increase in intracranial pressure. This can also lead to further complications like herniation of posterior fossa structures and brainstem compression, which leads to compression of respiratory and cardiac centers, leading to altered mental status or respiratory and cardiac failure. The treatment of fulminant cerebellitis should reduce intracranial pressure by reducing cerebellar swelling by administering immunomodulating agents like high-dose corticosteroids. Prompt treatment with steroids prevents surgical procedures like ventricular drainage, especially in patients with cerebellar edema and hydrocephalus [17].

## Conclusions

Diagnosing acute fulminant cerebellitis is very crucial in order to start early interventions in order to prevent fatal complications. Since there is a significant overlap of signs and symptoms between the benign and aggressive form of acute cerebellitis, MRI becomes the modality of utmost importance in patients with a high index of suspicion in order to avoid further irreversible complications later on.
